# Supplementary material for: Development and Evaluation of a Gatekeeper Training Program Regarding Anxiety about Radiation Health Effects Following a Nuclear Power Plant Accident: A Single-Arm Intervention Pilot Trial
Source: Int J Environ Res Public Health. 2020 Jun 26;17(12):4594. doi: 10.3390/ijerph17124594 (PMC7344952; doi:10.3390/ijerph17124594)
Supplement: Supplementary file 1 [file ijerph-17-04594-s001.pdf]

Supplementary S1. Entire training program for radiation health anxiety and other mental health issues (English translation)

[A. Presentation and group-work session (70 mins.)]

0. Aim for training: To acquire knowledge about anxiety toward radiation health effects and mental health issues (depression, suicide, alcoholism, and general anxiety) against the backdrop of the nuclear accident and to learn how to cope with residents with anxiety about radiation health effects or mental health issues through non-judgmental listening and referral to an appropriate resource or professionals.

1. Introduction: Anxiety about radiation health effects and mental health issues among residents while having some troubles in family and community relationships or economic issues and being forced to make a decision to return home.

1.1. Role of livelihood support counselors in Social Welfare Councils: Examples of answers from the survey of the gatekeeper training program needs among livelihood support counselors were "I am frustrated because I cannot give effective advice to residents with concerns about radiation only by listening non-judgmentally", "I want to learn the specific ways to cope with residents with mental health issues like alcoholism and suicidal thoughts", and "how to get cooperation with other expert facilities (not only "referring" but also communicating with residents with mental illness as a counselor)." Therefore, this training program was organized to teach livelihood support counselors about their role, for example, "listening non-judgmentally", "supporting gently", and "referring to an appropriate resource or professionals if necessary".

2. Specific coping skills for anxiety about radiation health effects and other mental health issues

2.1. Depression: Depression is a mood disorder subtype. It is characterized by the presence of symptoms such as depressive mood, loss of joyfulness for activities, and loss of interest in things that they used to enjoy, that continue for a couple of weeks.

2.1.1. Epidemiology of depression and psychological distress among evacuees in Fukushima.

Since the Fukushima Daiichi Nuclear Power Plant accident, the percentage of residents who are thought to have symptoms such as depression and anxiety in Japan has increased.

2.1.2. Encouraging early intervention for depressive states.

People who take a long time to receive treatment for depression tend to take longer to recover. Moreover, once you experience a depressive condition, it tends to become more likely to be repeated. Therefore, it is important to receive early support and prompt treatment in the early stage of depression.

2.1.3. Five-step principles of the Mental Health First Aid program (MHFA).

a) assess the risk of severity of depression and suicide risk: Protect the resident's privacy by choosing an appropriate time and place and do not put mental pressure on them. Carefully watch for signs of critical situations such suicidal thoughts or self-harm.

b) listen non-judgmentally: Avoid negative judgments, reactions, and cherished opinion like "don't be so depressed"

c) give reassurance and information:

d) encourage a person to get appropriate professional help: If the depressive state has continued for a couple of weeks and is affecting daily life, you should encourage the resident to get appropriate professional help

e) encourage other types of help and self-help: Those who feel that they are supported are more likely to recover.

2.2. Suicide: The majority of people who die by suicide have some kind of mental illness. Of the people aged 16 to 85 with depression, 4% have attempted suicide over the past year.

2.2.1. Epidemiology of suicide in the evacuating area in Fukushima

The male suicide rate in the evacuation area in Fukushima Prefecture increased immediately after the Great East Japan Earthquake and three years after the earthquake. The female rate increased in the two years after the earthquake. Therefore, it is necessary to support residents in the affected area after a large-scale disaster, keeping in mind that the suicide rate is likely to fluctuate.

2.2.2. Risk and protective factors of suicide:

Hopelessness, helplessness, a past history of attempted suicide or self-harm, mental illness, substance use problems such as alcoholism, unemployment, bereavement with family and important others, separation, isolation, absence of supporter, and male gender

2.2.3. Specific communication with residents who have suicidal thoughts

a) ask about the suicidal thoughts: You can ask a resident clearly "Are you thinking of suicide?" or "Are you thinking of dying?" Be confident and give peace of mind to them.

b) encourage a person to get appropriate professional help: Do not leave a person with suicidal thoughts and at high risk for suicide. Refer them to the appropriate resources or professionals as soon as possible.

2.3. Alcoholism: There are approximately 1.07 million suspected alcoholics

2.3.1. An outline of issues regarding alcoholism:

[Short term problems] Trauma, aggression, antisocial behavior, high-risk behavior, self-harm, and suicide

[Long-term problems] Alcohol and other drug use disorders, depression, anxiety, social issues, and physical health issues

2.3.2. The difference between heavy drinking and alcoholism:

The criterion of whether someone has alcoholism or not is not based on the amount of drinking but the fact that drinking causes problems or that they find it impossible to control their drinking even they understand the cause of their drinking problem. Therefore, alcoholism does not depend on the amount of drinking, but the degree of control over drinking. So, how much drinking corresponds to a heavy drinker? Moderate drinking is 20g of alcohol per day. Heavy drinking is 60 g/day.

2.3.3. The psychological factors of starting to drink alcohol after the Great East Japan earthquake:

The onset of drinking after the Fukushima Daiichi Nuclear Power Plant accident is associated with being male, younger, having dissatisfaction with sleep, and undergoing psychological distress following the disaster. Heavy drinking and psychological distress are risk factors for continued drinking among new drinkers. It should be noted that new drinkers who use alcohol to cope with disaster-related stress may be a suitable target group for disaster-related mental health services.

2.3.4. Five-step principles of the MHFA.

a) assess the risk of alcoholism: Talk with a resident when they are not drinking. Communicate supportively but do not show an attitude for tolerance with drinking alcohol. Assess the state of drinking.

b) listen non-judgmentally: Avoid threatening, conflicts, preaching, or labeling as "alcoholism."

c) give reassurance and information: Support a resident with alcohol problems while maintaining a favorable relationship. Understand the trans-theoretical model (stages of change) and ambivalence state.

d) encourage a person to get appropriate professional help: Wait patiently with compassion until a resident with alcohol problems acknowledges the necessity for professional help. What should continue is 1) the proposition of professional help, 2) a supportive attitude, and 3) communication of the risks of alcohol.

e) encourage self-help strategies: Confirm if there are people who can help (family, friends, self-help groups, etc.) and if a resident with alcohol problem has depression or anxiety.

2.4. Anxiety:

2.4.1. The outline of issues with anxiety: Everyone experiences anxiety. The level of anxiety varies from a little restlessness to a panic attack. However, in case of more severe, longer-lasting anxiety that interferes with work and relationships with family friends and colleagues, it is a problem.

2.4.2. Group work (10 mins.): Let us the list anxiety symptoms

(e.g.) Physical: Palpitations, chest pain, blush, fast, shallow breathing, shortness of breath, dizziness, headache, sweating, nausea, vomiting, diarrhea, muscle stiffness (especially neck and shoulders), shivering, and shivering

Psychological: Excessive fear, frustration, agility, and sleep disorders

Behavioral: Avoidance of certain situations, checking something many times, compulsion, avoidance of socializing, Use of alcohol or drugs

Thought: Excessive worry, decrease in concentration and memory, indecision, confusion

3. Key points for coping with anxiety about radiation health effects and other mental health issues

3.1. Skills for listening non-judgmentally:

[Attitude] Receptive, refrain from moral judgment, sincere, and empathetic.

[Linguistic skills] Listen without interruption, confirm understanding, give an agreeable response (but not excessively), summarize what the resident said

[Non-verbal skills (Body language)] Pay close attention to what a resident is saying, make eye contact, have an open posture, do not face the person directly, and sit and be polite.

3.2. Understanding of the trans-theoretical model (stages of change) and ambivalence state of suicidal thoughts and alcoholism among residents:

- The trans-theoretical model (stages of change) is a conceptual idea to change behavior through stages of "pre-contemplation", "contemplation", "preparation", "action", and "maintenance". You can work on a resident with mental health issues like suicide or alcoholism effectively by responding according to each stage.
- In an ambivalence state of suicide thoughts or alcoholism, a resident has opposite ideas at the same time. Be aware that there are two fluctuations in thought.

3.3. Association among depression, alcoholism and anxiety:

People with mental health problems often have multiple problems (e.g., depression, alcoholism, and anxiety) together. Be careful if the problems overlap.

3.4. Specific coping skills for residents with anxiety:

In an anxious state, people often overestimate their concerns and anxiety and underestimate their coping abilities and support from others. To reduce awareness of concerns and anxiety, be aware of coping abilities and support from others.

3.5. Specific coping skills for anxiety about radiation health effects (recommendation to measure the air radiation level or the radiation dose received by crops).

If a resident is concerned about the air radiation level in their living environment or the radiation dose received by crops, it is best to recommend that they measure the actual air radiation level or radiation dose received by crops using a radiation measuring instrument lent by the local government office.

3.6. Referral or signposting to an appropriate resource or professional:

Municipal health center (including public health nurses), disaster mental health center, hospital or doctor’s practice, radiation counselors, counselling telephone services, and self-help group for alcoholism

3.7. Self-help and self-care:

Self-help of the counselors themselves is also important. Sufficient sleep, avoiding excessive drinking alcohol, participation in leisure time and activities, listening to music and relaxing, and discussing between other counselors who have the same problem are means of doing this.

[B. Role-play session (30 mins.)]

1. Explanation of the scene-setting: (A 69-year-old woman lived in the ex-evacuation area. She and her husband have returned to the ex-evacuation area. However, her daughter's family did not choose to return home because of anxiety regarding radiation health effects. In addition, her husband has many troubles with alcoholism; therefore, she implied having some slight suicidal thoughts to a life support adviser.)

2. Role-play: (ordinal communication for evacuees) and group discussion

Counselor: Hello, long time no see. Are you getting used to being back here?

Resident: Hmm, it's nice to be back, but there are no neighbors, so it's completely different from my previous life. I tried to grow crops again. When I brought some crops to my son the other day, he said, "I don't need them because I have concerns about the radiation dose in these crops”. I'm kind of depressed. I thought “Is it useless to make it?”

Counselor: That is a shame.

Resident: Since the government have stated that it is safe here, the evacuation order has been, but, as my son says, do issues about radiation dose level still remain here?

Counselor: I think there is no problem because the government has told us that the current benchmark of the dose level of the crops is fine. By the way, I feel like you are a little bit thinner than before. How is your health condition?

Resident: Yeah, I have not had much of an appetite recently. Before the disaster, I use to live with my son's family, so I worked hard to prepare meals, but now, I live with only my husband, so I just wing it.

Counselor: I'm worried about that. Do you have any other problems?

Resident: To tell the truth, my husband has drunk alcohol for a long time, but recently, he has been drinking in the daytime. You know, he had lots of work and had to grow crops, so he had no time to drink, but he has had nothing to do since we came back here, and there are few people around us, so I think he is lonely and he is drinking all the time. He always gets a dirty mouth and angry while he is drinking. Actually, I don't really want him drinking alcohol, but he always gets angry if I tell him to stop drinking. So, to avoid getting him angry, I try to escape outside after buying only alcohol for him.

Counselor: Is that so? That sounds tough. Solving problems with alcohol is quite difficult. Has he ever battered you?

Resident: Hmm, sometimes …but I’ve already given up worrying about his behaviors while drinking.

Counselor: Are you alright? It sounds you lost appetite due to lots of stress. Is there anyone who you can talk to?

Resident: When my son used to live with us before the disaster, he could correct for his behaviors and always tell him "Don't drink too much". It seems he wanted to be modest because of my son's bride. But now, it is the only us, so it seems that he is just drinking when he wants to.

Counselor: You may have to manage his drinking behavior properly because you are the only family member who lives with him. If you manage his drinking well, your husband's problem may become a little better. By the way, is there anything else you care about? For example, are you sleeping well?

Resident: Somehow, I'm afraid to only have the two of us in my house. I feel that my heart is beating strongly because of anxiety. I'm worried that he will suddenly become rough and violent again. It has been a few months since our return here, but sometimes I can't sleep at all and I manage to do housework for him. However, even if I try to do housework for him, I worry that he will get drunk and behave terribly. So, I wonder if I should be gone (implying suicide).

Counselor: You think so seriously, don't you? How do you think about your future life?

Resident: I have intent to get along with my husband, but I think I can't help by myself sometimes.

Counselor: If so, I can refer you for a consultation with a public health nurse in the public health center. I think she can give advice about your physical condition and your family's drinking problems. If you want, I am familiar with her, so I can tell her about you in advance. Then, let's think together what we can do for your issues.

Resident: May I ask you later?

Counselor: Alright, no problem. I'll come back again later.

3. Role-play: (desirable communication for evacuees) and group discussion

Counselor: Hello, long time no see. How have you been after coming back here?

Resident: Hmm, it's nice to be back, but there are no neighbors, so it's completely different from my previous life. I tried to grow crops again. When I brought some crops to my son the other day, he said, "I don't need them because I have concerns about the radiation dose in these crops". I'm kind of depressed. I thought "Is there no point in making it?"

Counselor: That is a shame. Could you tell me more detail?

Resident: Since the government have stated that it is safe here, the evacuation order has been lifted, but, as my son says, do issues with the radiation dose level still remain here?

Counselor: Hmm. Certainly, some people still have concern about radiation exposure and the dose level, and the perception risk differ depends on the person. If the problem is just the radiation dose of crops, it will be measured at the local government office, and you can consult with a radiation counselor.

Resident: Thank you.

Counselor: By the way, I feel like you are a little bit thinner than before. How is your appetite? Can you eat meals properly?

Resident: I haven't had much of an appetite recently. Before the disaster, I use to live with my son's family, so I worked hard to prepare meals, but now, I live with only my husband, so I just wing it.

Counselor: I'm worried about that. Are you burdened with lot of stress?

Resident: To tell the truth, my husband has drunk alcohol for a long time, but recently, he has been drinking in the daytime. You know, he had lots of work and had to grow crops, so he had no time to drink, but he has had nothing to do since we came back here, and there are few people around us, so I think he is lonely and he is drinking all the

time. He always gets a dirty mouth and angry while he is drinking. Actually, I don't really want him drinking alcohol, but he always gets angry if I tell him to stop drinking. So, to avoid getting him angry, I try to escape outside after buying only alcohol for him.

Counselor: Is that so? That sounds tough. Solving problems with alcohol is quite difficult. Has he ever battered you?

Resident: Hmm, sometimes …but I've already given up worrying about his behaviors while drinking.

Counselor: Are you alright? In case of any physical harm to you, run away immediately and talk to the police. Is there anyone who you can talk to?

Resident: When my son used to live with us before the disaster, so he could correct for his behaviors and always tell him "Don't drink too much". It seems he was modest because of my son's bride, but now, it is only us, so he is just drinking when he wants to.

Counselor: That sounds tough. Let's think of something what we can do together. At times like this, I think it's hard to think of a good idea by yourself. Also, I don't have a good idea right away, but if we think about it together, something good might come up.

Resident: Well, thank you so much, that helps me. I appreciate that you have just listened to me. Recently, I have been afraid to be with only him in my house. I feel my heart is beating strongly because of anxiety. I'm worried that he will suddenly become rough and violent again. It has been a few months since we returned here, but sometimes I can't sleep at all and I manage to do housework for him. However, even if I try to do housework for him, I am worried that he will get drunk and behave terribly. So, I wonder if I should be gone (implying suicide).

Counselor: You think that seriously, do you? Have you felt so painful that you really want to die?

Resident: Not always, but I have thought about suicide several times.

Counselor: Have you thought about concrete ways or made plans about suicide?

Resident: Never a concrete way or plan so far.

Counselor: I see, but I worry about you. I can refer you for a consultation with a public health nurse in the public health center. I think she can give advice about your physical condition and your family's drinking problems. If you want, I am familiar with her, so I can tell her about you in advance. Then, let's think together what we can do for your issues.

Resident: May I ask you later?

Counselor: Alright, no problem. I'll come back again later.

[Comments: Points about coping with residents in ordinal communication]

- Don't make your own decisions, like" I think there is no problem because the government has told us that the current benchmark of the dose level of the crops is fine."
- You are better to look for something that you can do with a resident to help them stay healthy, rather than putting them under control of drinking, e.g., "You may have to manage his drinking behavior properly because you are the only family member who lives with him."

[Comments: Points about coping with residents in desirable communication]

- Showing the ability to listen to carefully, like" Could you give me more detail?"
- Confirming if there are any problems to address immediately and inform them of practical ways to do so, like "In case of any physical harm to you, run away immediately and talk to the police."

・ Asking if they have problems without any solution or there are any others to consult like "Is there anyone who you can talk to?"
・ Showing an attitude of thinking together (not trying to manage to advise) like "But if we think about it together, something good might come up".
・ Don't miss pessimistic remarks about the future and confirm the true meaning of "I wonder if I should be gone". Also, confirm suicide plannability and feasibility.
・ Refer or signpost the resident to an appropriate resource or professional in the case of possible suicide.

Supplementary S2. Entire training program for radiation health anxiety and other mental health issues (Japanese original)

[A. 講義およびグループワーク (70 分)]

0. 研修の目的: 【対象】避難指示解除後の地域で生活する住民からの相談を受けている生活支援相談員

【目的】原発事故や放射線不安を背景としたメンタルヘルスの問題(うつ、自殺、アルコール関連問題、不安）に対する知識を習得し、①傾聴、②対応、③関係機関につなげる一連の流れを学ぶ

1. はじめに: 避難区域内にもともと住んでいた人は、今回の原発事故やそれに伴う避難生活を余儀なくされた背景がある。また、原子力発電所事故による被ばくの不安を抱えた住民はもちろんのこと、避難した多くの住民も同じように、多かれ少なかれ放射線への不安を抱えている状況である。そのような背景の中、仕事や経済的な問題、家庭の問題、近所付き合い、睡眠が十分にとれなくなったなどの身体症状の出現など、避難生活によって生じた問題に加え、放射線の健康影響や飲料水・農作物への影響といった放射線そのものによる不安、さらには放射線不安の度合いの違いによって生じうる、避難生活を続けるか、帰還するかの判断を迫られる状況など、様々な形でストレスを抱えやすい状況になっている。

1.1. 生活支援相談員の役割: 昨年度実施したアンケート調査の結果から、メンタルヘルスの問題を抱えた住民を対応する際に、「傾聴するだけで良いのか」「具体的な対処の仕方を学びたい」「メンタルヘルスの知識を得たい」「相談を受けているが、自分自身も不安」といった意見があった。したがって、今回はメンタルヘルスの基礎知識について学ぶとともに、住民の声を傾聴し、寄り添い、一緒に進むかかわりを行いながら、現場で対応し、必要時に関係機関につなぐ行動がとれるように、研修を実施する。

2. 放射線被ばくによる健康への影響の不安やメンタルヘルス問題への具体的な対応

2.1. うつ病: うつ病とは気分障害の一種。「いつもと違う悲しい気持ちが消えない」「以前は楽しめた活動が楽しめず、興味がもてない」などの症状が２週間以上続くような状態。

2.1.1. 福島県の避難区域内の住民のうつ病と精神的苦痛（Kessler's 6, K6）の疫学

福島県では震災以降、うつ・不安などの症状と思われる人（K6　5 点以上）の割合が全国よりも多い状態が続いている。

2.1.2. うつ病に対する早期支援の重要性

うつ病の治療をうけるまでの期間が長い人は、回復までの時間がかかる傾向にある。また、一度うつ病状態を経験すると、その後もうつ病状態を繰り返しやすくなる傾向にある。したがって、最初のうつ病状態で早期に支援を行い、迅速かつ効果的な治療を確実に行うことが重要。

2.1.3. 対応の基本ポイント：メンタルヘルス・ファーストエイドの 5 つの基本ステップ「り・は・あ・さ・る」

a) 「り」声をかけ、リスクを評価し支援を始めましょう: プレッシャーをかけないよう、プライバシーが守られる時間や場所を選ぶ。危機的状況（自殺念慮・自傷行為）のサインを見逃さないようにする。

b) 「は」決めつけずに、話（はなし）を聞き、会話しましょう: ネガティブな判断や反応、「落ち込むことはないよ」とは言わない。

c) 「あ」安心（あんしん）につながる支援と情報を提供しましょう:

d) 「さ」専門家のサポートを受けるよう勧めましょう: 落ち込みが数週間にわたって続き、普段の生活に影響を与えている時は専門家の支援を勧める。

e) 「る」その他のヘルプやセルフヘルプ等のサポートを勧めましょう: 支えられていると感じている人の方がリカバリーに向かいやすい。

2.2. 自殺: 自殺を完遂した人の大多数は何らかの精神疾患を抱えていた。16〜85 歳のうつ病であった人のうち、4%の人が過去 1 年間で自殺しようとしたことがあった。

2.2.1. 福島県内の避難区域内の自殺の疫学

福島県内の避難区域内の自殺死亡率の動向では、震災以降、避難区域内の自殺死亡率が変動した。男性は震災直後と震災後 3 年経過した後、女性は震災後 2 年経過した後に上昇した。これまでの研究でも同様に、大規模災害後は地域の自殺死亡率が変動しやすいことを念頭に置いて、支援を行っていく必要がある。

2.2.2. 自殺の危険因子:

絶望感、無力感、自殺未遂、自傷の既往、精神疾患、アルコール等の物質使用問題、無職、失業、重要他者との死別、離別、孤立、支援者不在、男性

2.2.3. 自殺念慮のある人への対応

a) 本人に確認する:「自殺を考えているの？」または「死ぬことを考えているの？」とはっきり聞く。自信のある態度を示し、相手に安心感を与える。

b) 関係者につなげる: 自殺のリスクが高い人を一人にしない。すぐに専門家につなげる。

2.3. アルコール依存症・アルコール問題: アルコール依存症が疑われる者約 107 万人

2.3.1. : アルコール関連問題の概要

【短期的な問題】外傷、攻撃性、反社会的行動、リスクの高い行動、自傷、自殺

【長期的な問題】アルコールや他の薬物の使用障害、うつ、不安、社会的問題、身体的な健康問題

2.3.2. 多量飲酒とアルコール依存症の違い:

アルコール使用障害か否かの線引きは、飲酒量ではない。飲酒によって問題が生じているにもかかわらず、あるいは飲酒してしまうことで問題が生じてしまうことがわかっているのに、それでも飲酒を続けてしまう、否かである。したがって、その人にとっての程度の問題の部分がある。

節度ある適度な飲酒量:アルコール 20g/日、多量飲酒と定義される量: 1 日 60g、3 合程度以上

2.3.3. 東日本大震災後に新たに飲酒を開始した心理学的要因について:

福島県の避難区域住民の調査によると、（震災前は飲酒しておらず）震災後に飲酒するようになった人では、「精神的苦痛を抱えている」「睡眠の問題がある」「東日本大震災で、津波を経験した」「原発事故の爆発音を聞いた」「放射線被ばくによる健康不安がある」といった要因があった。これは、震災による体験などによる精神的苦痛や睡眠の問題に対し、『自己治療』的に飲酒行動をとった可能性が考えられる。アルコール問題を抱えた方の心理的背景として「何らかの精神的な苦痛を抱えた結果」であることを念頭に置くように。

2.3.4. 対応の基本ポイント：メンタルヘルス・ファーストエイドの 5 つの基本ステップ「り・は・あ・さ・る」:

a) 「り」声をかけ、リスクを評価し支援を始めましょう: 飲酒していないときに声かける。支持的に話すが、飲酒を続けることを容認しない。酔いの程度を評価する。

b) **「は」決めつけずに、話（はなし）を聞き、会話しましょう:** 脅かしたり対立したり、説教すること、「アル中」などのレッテルを貼ることは避ける。

c) **「あ」安心（あんしん）につながる支援と情報を提供しましょう:** 良好な関係性を維持して支援する。変化の段階モデルと両価性を意識して関わる。

d) **「さ」専門家のサポートを受けるように勧めましょう:**専門家の援助を必要とすることを認めるまで思いやりを持って、辛抱強く待つ。継続すべきことは、①専門家の援助を受けることを提案する、②支持的に接する、③アルコールによるリスクを伝える。

e) **「る」その他のヘルプやセルフヘルプ等のサポートを勧めましょう:** 助けてくれる人たちがいるか（家族や友人、自助グループなど）、うつや不安の問題があるかを確認

2.4. 不安:

2.4.1. 不安症状の概要: 誰でも不安を経験する。不安の程度は「ちょっと落ち着かない」から「パニック発作」といった状態まで、人によってさまざま。不安が問題となるのは、より重症で、長く続き、仕事や人間関係等を妨げるような場合。

2.4.2. グループワーク (10 分): 不安症状をリストアップする。

(例) 身体面: 動悸、胸痛、赤面、速い浅い息づかい、息切れ、めまい、頭痛、発汗、吐き気、嘔吐、下痢、筋肉のコリ（特に首、肩）、身震い、震え

心理面: 過度の恐れと心配、イライラ、短気、神経過敏、睡眠障害、悪夢

行動面: 特定の状況を避ける、何度も確認する、強迫行為、対人恐怖、人付き合いを避ける、アルコールや薬物を使う

思考面: 過度の恐れと心配、あれこれ思い悩む、集中力・記憶力の減退、優柔不断、頭が混乱する

3. 放射線被ばくによる健康への影響の不安やメンタルヘルス問題への対応のポイント

3.1. 傾聴スキル:

態度: 受容的に、道徳的判断は控える、誠実に、共感的

言語的なスキル: 遮らずに話を聞く、理解を確認する、相づち(多すぎずに)、まとめる

非言語的なスキル（ボディランゲージ）: 相談者が何をどのように言っているかに細心の注意を払う、アイコンタクト、開かれた姿勢で、真正面から対面しない、座って丁寧に対応する

3.2. アルコール依存症や自殺念慮のある人の変化の段階モデルと両価性を理解する:

変化の段階モデル:「無関心期」「関心期」「準備期」「実行期」「維持期」と段階を経て行動を変える、という考え方。それぞれの段階に応じた対応をすることで効果的に働きかけることができる。

両価性:メンタルヘルスの問題を抱えた人には、対極にある考えを同時に合わせ持つことが多い。例えば、アルコール依存があれば、やめなければならないことは十分わかっているけど、飲みたい」、自殺について考えている方でも「本当は生きたい」といった気持ちもあります。2 つの考えの揺らぎがあることに気づいてもらう。

3.3. うつ、アルコール関連問題、不安の関係:

メンタルヘルスの問題を抱えた人は、複数の問題を併せ持つことはよくあること。問題が重なっていないかを注意する。

3.4. 不安への対応の考え方:

不安状態では、 心配ごとを過大評価し、自分の対処能力や周囲からのサポートを過小評価していることが多い。心配ごとの認識を下げたり、自分で対処できることや周囲からのサポートを強く認識できる支援が役立つ。

3.5. 放射線不安についての対応 (空間放射線量や農作物の線量測定):

放射線は目には見えず、実態の分からないことが不安感を高めているとよく言われている。信頼度が不明な他者から間接的に知識を得るのではなく、相談者自身が測定し、自ら高いのか低いのかを判断してもらうことが不安の軽減に有効。放射線不安が強い場合は、不安内容を具体的に把握し、放射線相談員につなぐ。各市町村で、身近な生活空間の放射線量を測定する簡易測定器を貸し出ししている。

3.6. 関係機関につなげる（社会資源の活用）:

市町村保健センター（保健師）、こころのケアセンター、医療機関（かかりつけ医など）、放射線相談員、いのちの電話、自助グループ（断酒会）など

3.7. セルフヘルプ:

不安による悪影響を軽減する方法として「十分な睡眠をとる」「過度のアルコール摂取は控える」などがある。また、「余暇の時間や楽しい活動に参加する」「音楽を聴いてリラックスする」など自分にとってゆっくりできる方法を提案する。同じ課題を抱えている者同士で話し合うことも、大事なセルフケアの方法になる。

[B. ロールプレイ (30 分)]

1. 場面設定の説明: (現在、避難指示解除された地域に帰還し、夫(72 歳)と 2 人暮らし。震災前は、長男家族と同居。長男(47 歳)、長男の妻(46 歳)、子ども 2 人(18 歳と 15 歳)　※年齢は現時点。震災後は、中通りの B 市に避難していた。その時、A さん夫婦と長男家族は、別々の応急仮設住宅で生活していたが、近くで生活しており互いに交流はあった。避難指示解除後、A さん夫婦は、夫の「家督を全うしたい」という強い意向により、避難区域内の自宅を再建し帰還した。一方、長男家族は子どもの進学先や妻の勤務先の利便性、また放射線被ばくへの不安から、避難先で新たに新居を構え、生活する方向となった。A さんは帰還したものの、近隣住民はあまり戻っておらず、かつてのような地域交流はなく、話し相手もいない。震災前は自宅の畑で農作物を育てることを生きがいとしており、畑仕事を再開した。しかし、先日、長男家族に野菜を持っていくと、「放射線の心配があるからいらない」と言われ、非常にショックを受けた。夫はもともと問題飲酒があり、普段から気性が荒く、多量飲酒すると A さんへ暴言、暴力があった。長男家族と同居していた時は、長男が止めてくれたが、現在はそれもすぐにはできない。

（避難指示解除後 1 年目）あなたは生活支援相談員として顔なじみの A さんの自宅を訪問した。

2. ロールプレイ(ありきたりな対応)とグループディスカッション:

相談員: こんにちは、A さん。しばらくぶりです。こっちに戻ってきてどう、慣れました？

A さん: うーん、なんか戻ってきたのはいいけど、近所の人は誰もいないし、前の生活とは全然違うわね。畑仕事もまたやってみたけど、この前、息子のところに野菜持っていったら、「放射線がおっかないからいらない」って言われて。なんだかすごく落ち込んじゃってね。もう、作ってもしかたがないと思っちゃって。

相談員: それは残念でしたね。

A さん: やっぱり、息子が言うように、ここって国が大丈夫だって言っているから、避難指示も解除されたけど、やっぱり被ばくの問題ってまだあるのかしら？

相談員: どうですかね。でも国でも大丈夫って言っているから、大丈夫だと思いますよ。 そういえばなんだか前から比べると少しやせたような気がするけどいかがですか？

A さん: そうなの、最近食欲もあまりなくてね。前は息子家族と一緒だったから、頑張ってご飯も準備していたけど、今はお父さんと 2 人だけだから、適当に作っている感じ。

相談員: それは心配ですね。他に何か困っていることはないですか？

A さん: 実はね、お父さん、前からお酒は飲むんだけど、最近昼間から飲んでいることが多いのよ。ほら、前は農作業とかいろいろやること多かったから、飲む暇なんかなかったんだけど。こっちに戻ってからは特に何もすることがないし、周りにも人がいないもんだから、寂しいのか、なんだかずっと飲んでいるのよ。酔っぱらうと口も荒

くなって、本当は飲んでほしくないんだけど、止めるとかえって怒るし。だから、怒らせないように、お酒だけ買ってきてあげた後は、外で時間つぶすようにしているの。

相談員: そうなの？ それは大変ね。お酒の問題はなかなか難しいからね。でも旦那さん、あなたに手をあげたりしませんか？

A さん: たまにね。でもあきらめているの。

相談員: 大丈夫？それでいろいろストレスがかかって、食欲が落ちたりしているかもしれないですね。 だれか相談できる人はいますか？

A さん: 昔は息子が一緒だったから、息子がお父さんに対しても結構強く言えたからね。お嫁さんの目もある手前、少しは遠慮していたところもあったみたい。でも、今は私と 2 人だけだから、やりたい放題みたい。

相談員：それは、でも、今はあなたしかいないから、あなたがしっかりお酒の管理しなきゃダメかも。あなたがしっかり管理できれば、旦那さんのお酒ももう少し良くなるかも。ちなみに他に何か気になることないですか？ 例えば眠れないとか。

A さん: なんだかお父さんと 2 人きりになるのが怖くてね。心臓もドキドキしているし。また突然荒げるかが心配で。帰還してから、数か月なるけど、眠れない日もあったりで、家事をするのも精一杯。でも、せっかく家事をやったところで、お父さんとの二人暮らしで、酔っ払ってひどいことされるのであれば、いっそ私なんていなくなった方がいいのかしら。

相談員: そんなに深刻に考えることもあるんですね。これからの生活についてはどう思いますか。

A さん: ここで夫とうまくやっていきたいと思いますけどね。でも、自分だけではどうしようもないと思うこともあるんです。

相談員: それだったら、役場の保健師さんに相談してみたらいいかもね。自分の体調のことや、家族のお酒の問題について相談にのってくれますよ。保健師さんだったら私顔なじみだから、あなたのこと話してみても大丈夫？それから、一緒にできることも考えてみましょう。

A さん: お願いしようかな。

相談員: それじゃ、また来るわ。

3. ロールプレイ（望ましい対応）とグループディスカッション:

相談員: こんにちは、A さん。しばらくぶりです。こっちに戻ってきてどう、慣れましたか？

A さん: うーん、なんか戻ってきたのはいいけど、近所の人は誰もいないし、前の生活とは全然違うわね。畑仕事もまたやってみたけど、この前、息子のところに野菜持っていったら、「放射線がおっかないからいらない」って言われて。なんだかすごく落ち込んじゃってね。もう、作ってもしかたがないと思っちゃって。

相談員: それは残念でしたね。詳しく教えていただいてもいいですか？

A さん: やっぱり、息子が言うように、ここって国が大丈夫だって言っているから、避難指示も解除されたけど、やっぱり被ばくの問題ってまだあるのかしら？

相談員：どうですかね。確かに被ばくの不安を抱えている人はいるし、感じ方も人によって違うこともあるので。野菜の線量だけの問題だったら、役場でも測定してくれるし、相談ものってくれるみたいですよ。

A さん: どうもありがとう。

相談員：そういえばなんだか前から比べると少しやせたような気がするけど、食欲はどうですか？ご飯はちゃんと食べられていますか？

A さん: そうなの、最近食欲もあまりなくてね。前は息子家族と一緒だったから、頑張ってご飯も準備していたけど、今はお父さんと 2 人だけだから、適当に作っている感じ。

相談員: それは心配ですね。相当ストレスもかかっているんじゃない？

A さん: 実はね、お父さん、前からお酒は飲むんだけど、最近昼間から飲んでいることが多いのよ。ほら、前は農作業とかいろいろやること多かったから、飲む暇なんかなかったんだけど。こっちに戻ってからは特に何もすることがないし、周りにも人がいないもんだから、寂しいのか、なんだかずっと飲んでいるのよ。酔っぱらうと口も荒くなって、本当は飲んでほしくないんだけど、止めるとかえって怒るし。だから、怒らせないように、お酒だけ買ってきてあげた後は、外で時間つぶすようにしているの。

相談員: そうなの？ それは大変ね。お酒の問題はなかなか難しいからね。でも旦那さん、あなたに手をあげたりしていませんか？

A さん: たまにね。でもあきらめているの。

相談員：大丈夫？本当に危害があるならすぐに逃げて、警察とかに相談してくださいね。 だれか相談できる人は？

A さん: 昔は息子が一緒だったから、息子がお父さんに対しても結構強く言えたからね。お嫁さんの目もある手前、少しは遠慮していたところもあったみたい。でも、今は私と 2 人だけだから、やりたい放題みたい。

相談員: それは大変ね。何か一緒にできることを考えてみましょう。こういう時って、一人で考え込んでも良い考えって出てこないし、私がすぐに役立てるかわからないけど、一緒に考えていけば、何かいい方法が浮かぶかもしれないし。

A さん: そうね、どうもありがとう、助かるわ。話聞いてくれるだけでもありがたいし。最近、お父さんと 2 人きりになるのが怖くてね。心臓もドキドキしているし。また突然荒げるかが心配で。帰還してから数か月なるけど、眠れない日もあったりで、家事をするのも精一杯。でも、せっかく家事をやったところで、お父さんとの二人暮らしで、酔っ払ってひどいことされるのであれば、いっそ私なんていなくなった方がいいのかしら。

相談員: そんなに深刻に考えることもあるんですね。実際に死にたくなる位、辛いって感じているんですかね。

A さん: いつもではないけど、考えてしまうことはあるかな。

相談員: 具体的な方法を考えたり、計画したりは？

A さん: そこまではないかな。

相談員: でも、今のあなたの状況はとても心配があるから、役場の保健師さんに相談してみたらいいかもね。自分の体調のことや、家族のお酒の問題について相談にのってくれますよ。保健師さんだったら私顔なじみだから、あなたのこと話してみても大丈夫？それから、一緒にできることも考えてみましょう。

A さん: お願いしようかな。

相談員: それじゃ、また来るわ。

[解説: ありきたりな対応についてのポイント]

・ 「でも国でも大丈夫って言っているから、大丈夫だと思いますよ」といった自分の考えで決めつけずに、丁寧に相手の話を聴く。

・ 「あなたがしっかりお酒の管理しなきゃダメかも。あなたがしっかり管理できれば、旦那さんのお酒ももう少し良くなるかも。」と、この場面では飲酒のコントロールを家族の責任に負わせるよりも、一緒にできることや家族自身が健康的に過ごすことのできることを探していく。

[解説: 望ましい対応についてのポイント]

・ 「詳しく教えていただいても大丈夫？」と丁寧に話を聴く姿勢を示している。

・ 「本当に危害があるならすぐに逃げて、警察とかに相談してくださいね。だれか相談できる人はいますか？」とすぐに対応が必要な問題があるか否かを確認し、実用な対応方法をすぐに伝えている。

・ 問題を一人で抱えこんでいないか、助けを得ることができる人がいるか聞いている。

- 「一緒に考えていけば、何かいい方法が浮かぶかもしれないし。」と一緒に考え寄り添う姿勢を示している（無理にアドバイスをしようとしていない）。
- 「いっそ私なんていなくなった方がいいのかしら。」といった将来に悲観的な発言は見逃さず、「死にたくなるくらいつらい」出来事なのか、その真意を確認している。また、自殺の計画性、実行可能性も確認している。その上で、専門家につなぐことができている。
- さらに、その際、専門機関では何ができるのか、情報提供をしている。
